# A Bioinspired Soft Robot Combining the Growth Adaptability of Vine Plants with a Coordinated Control System

**DOI:** 10.34133/2021/9843859

**Published:** 2021-10-22

**Authors:** Pengchun Li, Yongchang Zhang, Guangyu Zhang, Dekai Zhou, Longqiu Li

**Affiliations:** State Key Laboratory of Robotics and Systems, Harbin Institute of Technology, Harbin, Heilongjiang 150001, China

## Abstract

Tip-extending soft robots, taking flexible film or rubber as body material and fluid pressure as input power, exhibit excellent advantages in constrained and cluttered environments for detection and manipulation. However, existing soft continuum robots are of great challenges in achieving multiple, mutually independent, and on-demand active steering over a long distance without precise steering control. In this paper, we introduce a vine-like soft robot made up of a pressurized thin-walled vessel integrated with the high controllability of a control system with multiple degrees of freedom in three dimensions. Moreover, steering and kinematic models to relate the steering angle and robot length to the location of the robot tip are provided, and a dynamic finite element model for analyzing the motion of the spatial consecutive steering is established. We demonstrate the abilities of disinfection of the robot moving in a long and tortuous pipeline and detection in a multi-obstacle constrained environment. It is established that the robot exhibits great advantages in active consecutive steering over a long distance, high controllability in completing more complex path planning, and significant ability of carrying operational tools for ventilation pipeline disinfection and multi-obstacle detection. The bionic soft robot shows great promise for use in environment sensing, target detecting, and equipment servicing.

## 1. Introduction

Soft robots have great advantages, such as dexterous movement behavior, unprecedented adaptation, and extremely high security, for inspection and exploration work in constrained, uncharted, and cluttered environment [1–6]. Among the different types of soft robots, soft continuum robots, which are designed based on the principle of vine growth, have attracted significant attention due to the following unique advantages: (1) pneumatic eversion to move independent of the environment, (2) flexible feature to mitigate the damage, and (3) infinite extension and unlimited degrees of freedom to expand workspace [7–12]. However, their continuum nature introduces various challenges for modeling and controlling [13, 14]. For instance, their poor controllability restricts their motion to relatively simple path planning, which is not suitable for complex environments. Therefore, enhancing the motion controllability of soft robots is a top priority in soft robotic research. Hawkes et al. used prefabricated latches that pinched the everting body to achieve bending by breaking latches using pressure, and various demonstrations were completed during their research [8]. Greer et al. installed series pneumatic artificial muscles (sPAMs) along the robot body to investigate the navigation of the robot by inflating the contraction of sPAMs. They established that the robot could be successfully steered to a specific destination [15]. Do et al. used the values contained in the pouches to create locally compliant regions, and then, two cables were used to produce a large number of possible shapes [16]. However, in Hawkes et al., the pressure can only work on the latch at the tip with a long response time to reach the broken pressure over a long distance, and the sPAM scheme is limited to a simple path because it is impossible for one pneumatic artificial muscle to control multiple steering accurately. In addition, it is difficult for the tension of the cable to overcome the friction of multiple steering and then be transmitted to the tip with a long length for the robot. Consequently, achieving multiple, mutually independent, and on-demand active steering over a long distance is significantly challenging for the motion control of soft continuum robots.

Because vines lack a control system similar to animals, designing the soft continuum robot simply based on vine is limited in on-demand active steering ability. To overcome this knowledge gap, we provided a control system for biomimetic soft continuum robots imitating vines to avoid obstacles with a swift response actively. The inverted material passing through the center is the “waiting cell” for tip growth [8, 9, 17, 18], and the release of the main body stored between an electromagnet unit causes asymmetric growth like vines expanding their degree of freedom. To improve the controllability of the robot, electromagnets are controlled by using chip modules with different signal addresses to recognize and navigate the signal to the corresponding actuators, which realizes the decoupling control of the actuators, making the controllable continuous steering possible. Compared with the latches in Hawkes's work, the scheme introduces a multi-joint space steering design with up to eight states per joint and further realizes the simultaneous control of multiple joints not just the tip joint, which realizes more complex shape configurations and variable path planning; the attached circuits of control system provide higher control reliability and shorter response time for the process of steering; these advantages make the robot promise for a broad range of applications.

The remainder of this paper is organized as follows. In Section 2, we propose the basic design of a bionic soft robot as well as its manufacturing method. In Section 3, we establish the steering and kinematic models to relate the steering angle and robot length to the location of the robot tip. In Section 4, we experimentally verify the capability of the complex path planning of the robot and demonstrate possible applications for disinfecting ventilation pipeline and detecting multi-obstacle environment.

## 2. Design

**Figure 1:**
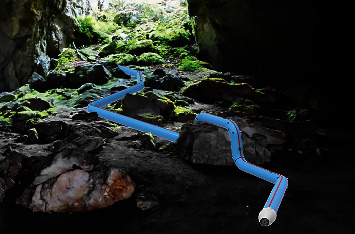
The conceptual design of the bioinspired soft robot.

The tip-extending, signal-controlling soft robot draws inspiration from the vine growth system. The eversion motion from the tip and asymmetric growth caused by the electromagnet units in the movement system imitates the motion behavior of natural vines to avoid obstacles and trace the target, and the decoupling control and centralized management of the steering actuators in the control system makes the movement complicated but coordinated. Therefore, we proposed a bioinspired soft robot combining the growth adaptability of vine plants with a coordinated control system, as shown in Figure 1.

In the motion system of the robot, the design of the tip growth and directional navigation device has attracted significant efforts in previous research [15, 16, 19–22]. The growth principle of pneumatic eversion is used in this study. The diameter of the main body made of thin-walled polyethylene is 76 mm. The electromagnet steering unit, which is inspired by latches and composed of a circular plate and a miniature electromagnet for storing part of the robot body, is attached to the robot along the robot body using double-sided tape. The electromagnet with a size of *φ*12 mm × 12 mm can provide a magnetic force of more than 10 N. This force is generated when power is off and disappeared when power is on, which effectively prevents the overheating of an ordinary electromagnet with a small size when it is energized for a long time.

**Figure 2:**
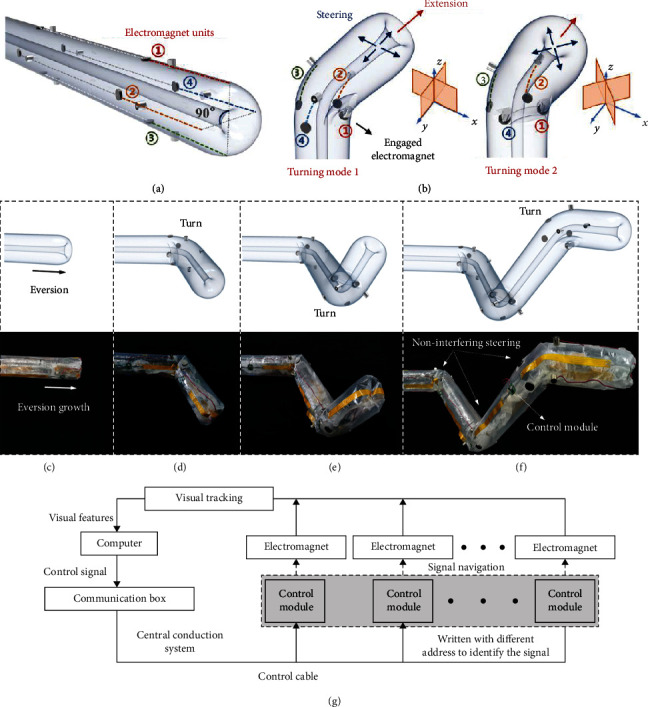
Principle of design and control for continuous steering. (a) Four series electromagnet units are uniformly distributed at 90° along the body of the soft robot. (b) The electromagnet units release the stored body to make the asymmetric lengthening, and the steering can be held by one electromagnet unit such as ① or two adjacent electromagnets such as ① and ④, corresponding to two different turning modes. (c) The robot is in a state of waiting for growth. (d) The electromagnets release the stored body through turning mode 1 and the robot steers to right. (e) Using turning mode 2, the robot steers upward. (f) The robot turns again to create three noninterfering steering at the spatial level. (g) Control block diagram of the robot.

As shown in Figure 2(a), the robot is composed of a backbone and four series of electromagnet units uniformly distributed at 90° in the cross-section. The steering occurs by the separation of the electromagnet and circular plate, and it can be controlled by a single electromagnet or two adjacent electromagnets. That is, there are two different turning modes with the steering in eight directions in space, as shown in Figure 2(b). The stored body is released when the electromagnet is energized, resulting in a difference in length between the two sides of the body, which simulates the variance in cell growth of the vine when it bends. The straight line at the electromagnet unit occurs when electromagnets on the same joint are opened synchronously or not at all. Figures 2(c)–2(f) show an active continuous steering process for completing a spatial path planning. Relative to the robot body with a few meters or even dozens of meters, the size of the electromagnet has a negligible effect on its flexibility.

In the control system of the soft robot, the decoupling electromagnet units are controlled by chip modules written with different signal addresses, whose function is to conduct the current for electromagnets when the received control signal is consistent with the address of the module. The module is integrated with the MAX232 and MK02FNVFM10 chips to receive and process the signal, respectively, and the modules are connected using a flexible printed circuit wire whose flexibility is almost similar to that of the robot body. The working process of the control system is shown in Figure 2(g). First, the environmental feature is transmitted to the computer through the vision system, which generates a control signal and sends it to the signal cable via a communication box. The control module with the same address as the signal controls the electromagnet unit to release the stored body, which realizes the precise navigation of control signals to actuators. The states of the actuators are reflected in the visual window by a visual feedback system and recorded on the computer. The design of the control system enables a human operator to control all the actuators of the robot via a simple interface, which realizes decoupling control and integrated management of steering actuators.

## 3. Modeling

### 3.1. The Steering Model

**Figure 3:**
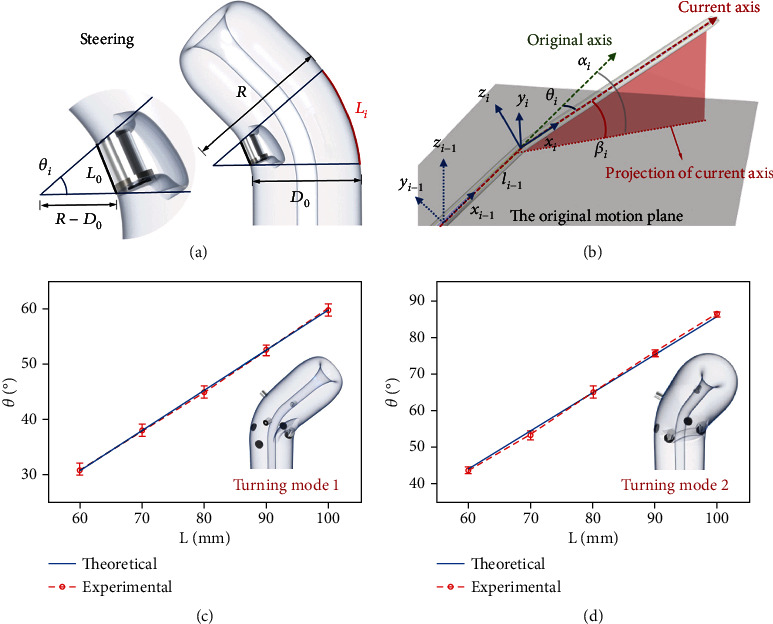
Models of the soft robot. (a) Schematic diagram of the steering kinematic model. (b) Kinematics with configuration parameters of the robot during steering. (c) Theoretical and experimental curves of turning mode 1. (d) Theoretical and experimental curves of turning mode 2.

To control the motion trajectory of the tip-growing soft robot, a steering model relating the bending angle to the length of the stored body must be established. The geometric relation of the steering shown in Figure 3(a) is used to express the steering model as follows:

(1) $$\begin{array}{c} L_{i}=\theta_{i}R,\\ \sin\frac{\theta_{i}}{2}=\frac{L_{0}/2}{R-D_{0}}, \end{array}$$

where *L*_*i*_ is the length of the body stored between the electromagnet and the circular plate, *θ*_*i*_  is the bending angle, *R*  is the radius of curvature of the steering, *L*_0_  is the thickness of the electromagnet unit after absorption, and *D*_0_  is the projection distance between the absorbing electromagnet unit and the outer edge of the moving plane. Further,  *L*_0_ and *D*_0_ are parameters that can be measured from the model. Therefore, we can adjust the length of the stored body to achieve the required bending angle.

### 3.2. The Kinematic Model

Various methods, such as the piecewise constant curvature [23, 24], classical D-H coordinate system [25, 26], and finite element [27], have been used to describe the kinematic model of soft robots. Hannan et al. established kinematic models of soft robots using the piecewise constant curvature method. However, this method is not applicable for soft robots with small bending curvature [20]. The bending posture of the flexible rod in this study is similar to that of the joint and connecting rod; therefore, the classical D-H method is used in this study. Two issues that differ from rigid robots are as follows:

1. Because the length of the flexible rod of the soft robot is adjustable compared to that of a rigid robot, the length of the rod, *l*_*i*_, becomes a variable rather than a constant value in the kinematic model for the rigid robot
2. In addition to the variable of bending angle, *θ*_*i*_, a novel variable of bending plane angle, *α*_*i*_, is introduced considering the characteristics of infinite degrees of freedom

Detailed information for the modeling of a soft robot steering in a free environment is shown in Figure 3(b). The following relationship can be obtained when |*α*_*i*_| ≠ *π*/2:

(2) $$\begin{array}{c} \cos\beta_{i}=\frac{\cos\theta_{i}}{\cos\alpha_{i}}, \end{array}$$

where the unknown parameter *β*_*i*_, which is difficult to measure directly, can be obtained using Equation (2). The parameters of the kinematic model of the robot are {*θ* *α* *l*}, which simplifies the complexity of the parameter setting. However, when |*α*_*i*_| = *π*/2, |*θ*_*i*_| = *π*/2, as a special case, *β*_*i*_ must be measured from the model.

The transformation in the forward kinematic model between the joints can be realized via the following steps: the center of the base coordinate system is translated *l*_*i*−1_ along axis *x*_*i*−1_, rotated *α*_*i*_ around axis *z*_*i*−1_, and then rotated *β*_*i*_ around axis *y*_*i*−1_. The homogeneous transformation matrix, *T*, from the base coordinate system to the terminal coordinate system can be manipulated as follows:

(3) T=ii−1Transli−1,0,0 Rotzi−1,αi Rotyi−1,βi,T=100li−1010000100001ii−1 cosαi−sinαi00sinαicosαi0000100001 cosβi0sinβi00100−sinβi0cosβi00001,T=cosαicosβi−sinαicosαisinβili−1sinαicosβicosαisinαisinβi0−sinβi0cosβi00001ii−1.

After obtaining the relationship between the length of the stored body and the bending angle using Equations (1) and (2), *T* can be computed via simple matrix manipulation. The matrix relating the arc-space parameters to the position and orientation of the robot tip can be solved.

## 4. Results and Discussion

To verify the steering kinematic model, we set five different lengths of the robot body stored in the electromagnet unit, generating five corresponding angles. To avoid measurement errors during the experiment, we used a protractor to measure the steering angle of the axis of the turning body five times and calculated the average value. The steering angles as a function of *L*_*i*_ for turning modes 1 and 2 are shown in Figures 3(c) and 3(d), respectively. It can be observed that the theoretical angle is highly consistent with that obtained experimentally.

**Figure 4:**
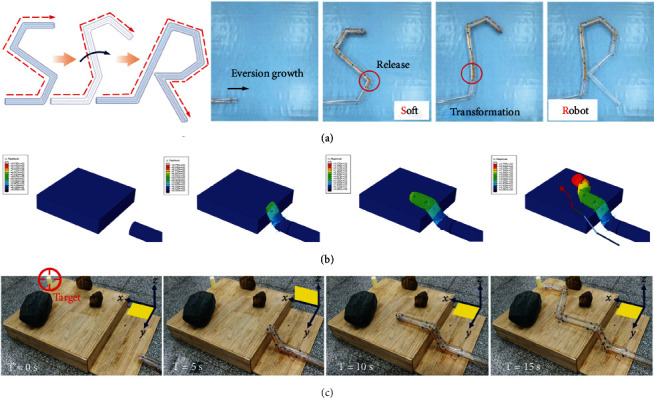
Simulation and demonstrations of models. (a) The deformation process of the initials “Soft Robot” from S to R. (b) The motion simulation of the soft robot to climb the steps to change the working plane. (c) Actual motion demonstration corresponding to simulation. The robot reaches the step and turns upward to climb on the step with multiple obstacles, after changing the work plane, the robot plans the path on the new plane to avoid all obstacles and finally reaches the target.

The robot motion under the control of the target navigation system enables the robot to complete complex path planning in a more coordinated manner.  Figure 4(a) shows the transition process of the initials of “Soft Robot” from S to R for a single soft robot. The robot grows from the tip through eversion and steers at the joints through the electromagnet steering units. The first four joints are designed using four steering angles of 60°, 80°, 80°, and 60° because they can generate a neat shape of “S.” To transform S into R, it is necessary to first straighten part of the tortuous path of S, which can be realized by releasing all the electromagnet units on the second joint. Thereafter, the neat shape of R is generated by steering three times at angles of 60°, 35°, and 90°. All the steering angles can be achieved because the length of the stored body can be predesigned using the verified steering model. The confirmatory demonstration of seven consecutive turns illustrates the feasibility of the motion and control system and the complex configuration capabilities of the resulting soft robot.

We established a dynamic finite element model to analyze the motion of the spatial steering robot crawling on the stairs, as shown in Figure 4(b). An inverted thin-walled body was designed with a diameter of 76.4 mm and a total length of 1150 mm. A connector force of 10 N was used to simulate the magnetic attraction force in the electromagnet units, whereas a fluid cavity with an inflation pressure of 0.01 MPa was used to simulate an inflatable air chamber. During the inflation simulation, the internal nongrowing body is everted at the apex of the robot, whereas the corresponding connector force on the turning path is released at the joints, which realizes the continuous spatial movement of climbing a stair stably and smoothly.

As shown in Figure 4(b), the displacement change of the main body gradually decreases from the apex to the initial position, which is in line with the actual motion of the continuous eversion growth driven by air pressure. The noninterfering steering caused by the release of the stored body is consistent with the design expectation of the steering scheme. This simulation theoretically verified the rationality of eversion growth from the apex and the spatial steering mechanism controlled by the four series of electromagnet units. Corresponding to the simulation, Figure 4(c) shows the spatial motion of the soft robot on climbing stairs, avoiding obstacles, and reaching the target on another working plane experimentally. The robot with a length of 2.3 m moves on the *XY* plane and encounters a stair with a height of 20 cm. According to the kinematic model, the robot turns upward by turning mode 2 at an angle of 80° in the *XZ* plane to climb on the stair. Thereafter, the robot steers twice to avoid obstacles on the new *XY* plane and reaches the target with high position accuracy.

**Figure 5:**
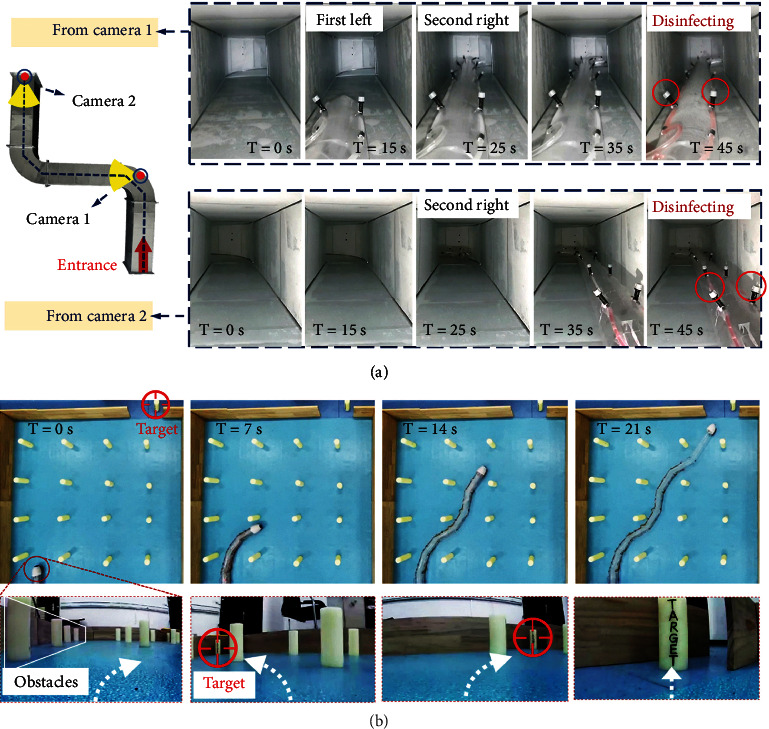
The pipeline disinfection by soft robot and the detection experiment in multi-obstacle environment. (a) The robot attached with two series of sterilizing nozzles enters the ventilation pipeline for disinfection. *T* = 15 s, the robot steers left at the first bend of the pipeline and steers right at the second at *T* = 25 s to comply the tortuous path of the pipeline; *T* = 35 s, the robot runs through the pipeline, and then, the disinfecting valve is opened to disinfect at *T* = 45 s without missing any corner. (b) The soft robot navigates to the target by using a camera at the tip. *T* = 0 s, the left side of the view is full of obstacles, so the robot turns right to avoid; *T* = 7 s, the camera finds that the target is located to the left of the view, so it steers left; *T* = 14 s, the target is to the right and it steers right to approach to; while the target is in the center of the view, the robot completes the path selection and reaches the target at *T* = 21 s.

Figure 5 demonstrates the capacity of the soft robot as a carrier for disinfection and detection in challenging constrained environments. Figure 5(a) shows the soft robot moving in a pipeline for disinfection. The length of the soft robot is more than 5 meters. Two disinfection hoses installed with atomizing nozzles at an interval of 30 cm are attached to the robot body and everted from the apex. In contrast to conventional disinfection equipment with tail propulsion, the soft robot grows and navigates on the apex, and this makes the disinfection robot adapt to the tortuous path of the ventilation pipeline. The passive deformation owing to its flexible characteristics and the active steering controlled by the electromagnet units relieves the restriction of the robot movement in the narrow pipeline environment; thus, the robot can pass through the inside of the pipeline. In addition, the high-density atomizing nozzle attached to the robot spine ensures a high efficiency of disinfection without missing any corner. This disinfection equipment is necessary for disinfection applications in hospital ventilation pipeline, specifically in the context of the COVID-19 outbreak in early 2020. Figure 5(b) demonstrates the implementation of the detection of the soft robot in a multi-obstacle constrained environment. The soft robot is extended through an obstacle array and it reaches the specified target through real-time path selection. A camera that feeds the environmental characteristics back to the computer to allow visual sensing is mounted on the apex of the robot. Enabled by consecutive steering, the robot continuously searches for the target while turning in advance to avoid obstacles in the view of the tip camera. The highly flexible and controllable apex of the robot achieves detection without contact with the environment, ensuring reliable adaptation to constrained environments with various obstacles that may destroy the robot body, such as sharp or overheated objects. This indicates great potential of the soft robot in postdisaster rescue or detection of space station failure.

## 5. Conclusion

In this study, we proposed a novel bioinspired continuum soft robot with multiple degrees of freedom in three dimensions by combining the growth adaptability of vine plants with a coordinated control system. Four series of electromagnet steering units, which are controlled by the corresponding chip modules written with different signal addresses to identify and navigate signals, are arranged along the body of the tip-growing robot to navigate its tip direction. Compared with Hawkes's work, the above design introduces a multi-joint space steering design and further realizes the simultaneous control of multiple joints not just the tip joint with higher control reliability and shorter response time for the process of steering. We also developed a mathematical steering model and a kinematic model based on the D-H method to relate the steering angle and robot length to the location of the robot tip. Experiments were performed, and it was established that the proposed model is quite accurate to predict the position showing by precisely controlling the transition for different shapes as required. Additionally, it was found that the robot shows great advantages in active consecutive steering over a long distance provided by electromagnet steering units, high controllability realized by completing more complex path planning, and great ability of carrying operational tools such as a camera on the apex or disinfection hoses along the body in an unpredicted challenge environment for ventilation pipeline disinfection and multi-obstacle detection. The bionic soft robot shows great promise for applications in environment sensing, target detecting, and equipment servicing. Future work will be devoted to developing new actuators to realize reversible bendability on the premise of ensuring high controllability and long growth distance.

## Supplementary Materials

Supplementary 1 Supplementary Movie S1: the deformation from S to R.

Supplementary 2 Supplementary Movie S2: the motion simulation of climbing on a step.

Supplementary 3 Supplementary Movie S3: actual motion demonstration of climbing on a step.

Supplementary 4 Supplementary Movie S4: the disinfection in the tortuous pipeline.

Supplementary 5 Supplementary Movie S5: the detection in multi-obstacle environment.
